# 3D printing of radioactive phantoms for nuclear medicine imaging

**DOI:** 10.1186/s40658-020-00292-0

**Published:** 2020-04-22

**Authors:** Tilman Läppchen, Lorenz P. Meier, Markus Fürstner, George A. Prenosil, Thomas Krause, Axel Rominger, Bernd Klaeser, Michael Hentschel

**Affiliations:** Department of Nuclear Medicine, Inselspital, Bern University Hospital, University of Bern, CH-3010 Bern, Switzerland

**Keywords:** 3D printing, SPECT, Technetium-99m, Resin monomer, Printer ink, Phantom, Scanner validation

## Abstract

**Background:**

For multicenter clinical studies, PET/CT and SPECT/CT scanners need to be validated to ensure comparability between various scanner types and brands. This validation is usually performed using hollow phantoms filled with radioactive liquids. In recent years, 3D printing technology has gained increasing popularity for manufacturing of phantoms, as it is cost-efficient and allows preparation of phantoms of almost any shape. So far, however, direct 3D printing with radioactive building materials has not yet been reported. The aim of this work was to develop a procedure for preparation of ^99m^Tc-containing building materials and demonstrate successful application of this material for 3D printing of several test objects.

**Method:**

The desired activity of a [^99m^Tc]pertechnetate solution eluted from a ^99^Mo/^99m^Tc-generator was added to the liquid 3D building material, followed by a minute amount of trioctylphosphine. The resulting two-phase mixture was thoroughly mixed. Following separation of the phases and chemical removal of traces of water, the radioactive building material was diluted with the required volume of non-radioactive building material and directly used for 3D printing.

**Results:**

Using our optimized extraction protocol with trioctylphosphine as complex-forming phase transfer agent, technetium-99m was efficiently transferred from the aqueous ^99^Mo/^99m^Tc-generator eluate into the organic liquid resin monomer. The observed radioactivity concentration ratio between the organic phase and the water phase was > 2000:1. The radioactivity was homogeneously distributed in the liquid resin monomer. We did not note differences in the 3D printing behavior of the radiolabeled and the unlabeled organic liquid resin monomers. Radio-TLC and SPECT studies showed homogenous 2D and 3D distribution of radioactivity throughout the printed phantoms. The radioactivity was stably bound in the resin, apart from a small amount of surface-extractable radioactivity under harsh conditions (ethanol at 50 °C).

**Conclusions:**

3D printing of radioactive phantoms using ^99m^Tc-containing building materials is feasible. Compared to the classical fillable phantoms, 3D printing with radioactive building materials allows manufacturing of phantoms without cold walls and in almost any shape. Related procedures with longer-lived radionuclides will enable production of phantoms for scanner validation and quality control.

## Background

Tomographic medical imaging procedures have become indispensable in modern healthcare, and their importance is continuously increasing as the techniques become more advanced, and the application space is growing. Among the tomographic imaging modalities, in combination with a suitable radiotracer, the nuclear imaging procedures positron emission tomography (PET) and single-photon emission computed tomography (SPECT) allow molecular imaging of physiological processes and pathological deviations at the molecular level. Following the discovery of numerous new radiotracers for various molecular targets and disease markers in the past decades, a growing number of these radiotracers have found their way into the clinical routine [1–4]. For evaluation and interpretation of imaging data, reliable standards for image acquisition, reconstruction, processing, and quantification are of utmost importance, in particular to assure the comparability of data acquired on different scanners or in different medical centers [5]. This issue becomes even more urgent in view of the growing number of therapeutic radiopharmaceuticals routinely applied in the clinic. The newly emerging theranostic approaches in nuclear medicine require proper dosimetry calculations [6], which in turn depend on the availability of accurate and reliable imaging data.

To ensure reliable standards, nuclear imaging systems are generally validated under different circumstances using physical phantoms. Traditionally, these phantoms are hollow cylinders containing hollow spheres, which are usually manufactured by molding techniques and then filled with radioactive liquids. While the geometric complexity of phantoms produced by conventional molding methods is somewhat limited, recently introduced 3D printing techniques allow preparation of phantoms of almost any shape, including anthropomorphic phantoms with fine structures, such as different organs and highly irregular tumor lesions [7, 8]. So far, 3D printed phantoms have mainly been used for computed tomography (CT), followed by magnetic resonance imaging (MRI), and ultrasound (US). Only a small number of reports have described 3D printed phantoms in conjunction with the nuclear imaging techniques PET and SPECT, and usually the radioactive material is introduced into the empty 3D printed phantom in the form of radioactive solutions, see Filippou et al. [9] and Valladares et al. [10] for recent reviews. Embedded into a radioactive background, the walls of such filled phantom inserts remain “cold,” i.e., they are devoid of radioactivity. This approach introduces background dependent bias in PET phantom measurements [11]. Cold walls affect mostly small inserts, which then become poorly comparable to lesions in patients that do not have cold walls. 3D printable radioactive materials would allow printing of phantoms and small phantom inserts of any shape without cold walls. Surprisingly, despite the huge potential, there are only few reports on printing of radioactive materials, and all of them are limited to 2D printing on paper sheets using a standard inkjet printer and a mixture of aqueous radioactive solutions mixed with the printer ink. The papers with 2D printed radioactive patterns were then stacked together in fixed distances, separated by thin layers of polystyrene or polymethylmethacrylate (PMMA) to build 3D sandwich phantoms for SPECT (based on technetium-99m) [12–15] or PET (based on fluorine-18) [16].

Following the successful 2D printing using radioactively spiked ink, direct printing of radioactive material in a 3D printer may seem straightforward. There are, however, a number of complicating issues. First of all, there are the inherent challenges associated with printing of radioactive materials, such as contamination of printer parts, tools, washing solutions, and potential leakage and spills, which are in particular problematic when utilizing radionuclides with long half-lives. Most importantly, however, there are currently no established methods for incorporating radionuclides into the liquid resin monomers used for 3D printing. Simple mixing of the liquid resin monomer with radioactive solutions is not possible, as most radionuclides are present in the form of aqueous solutions, which are not miscible with the organic acrylate-based liquid resin monomers. Consequently, to the best of our knowledge, radioactive phantoms printed on a 3D printer by using liquid resin monomers (“3D printer ink”) spiked with radionuclides have not yet been reported.

In this work, we present a novel method for the preparation of ^99m^Tc-containing organic liquid resin monomers and demonstrate for the first time successful application of such a radioactive building material for 3D printing of several test objects. Although the current proof-of-concept study is limited to the radionuclide technetium-99m, we foresee that the methodology can be adapted to a broad range of different 3D printing materials and radionuclides and will ultimately lead to widespread use of this technology in the manufacturing of radioactive phantoms.

## Methods

### General information—devices, materials, and radioactivity measurements

The 3D phantoms described in this technical note were prepared on a ProJet® 1200 3D Printer (3D Systems, Inc., USA) operating with enhanced LED Digital Light Processing (DLP) technology. The net build volume of the ProJet® 1200 3D Printer is 43 × 27 × 150 mm^3^ (xyz), the native resolution (xyz) 0.056 mm (effective 585 dpi), the layer thickness 0.03 mm, and the vertical build speed 14 mm/h [17]. The build material (liquid resin monomer) “VisiJet FTX Green” is a UV-curable plastic with a density of 1.04 g/mL (25 °C). It is an organic mixture containing 40–55% of triethylene glycol diacrylate, 15–25% of tricyclodecane dimethanol diacrylate, and 1.5–2.5% of the photoinitiator phenylbis (2,4,6-trimethylbenzoyl)phosphine oxide [18].

All chemicals were purchased from commercial suppliers and used as received. Trioctylphosphine (97%), trimethyl orthoformate (99%), *n*-butyl acetate (≥ 99.5%, p.a.), 0.1 N hydrochloric acid, and 0.1 N sodium hydroxide solution were from Sigma-Aldrich/Merck KGaA (Darmstadt, Germany), 2-propanol (≥ 99.8%, p.a.) from Carl Roth GmbH & Co. KG (Karlsruhe, Germany), and 96% ethanol from Hänseler AG (Herisau, Switzerland).

[^99m^Tc]Pertechnetate in 0.9% aqueous NaCl was eluted from an Ultra-Technekow FM ^99^Mo/^99m^Tc-generator (b.e. imaging AG, Schwyz, Switzerland). Extraction of radioactivity from the ^99^Mo/^99m^Tc-generator eluate into the resin was performed in polypropylene (PP) microcentrifuge tubes. A Spectrafuge™ 24D microcentrifuge (Labnet International, Inc.) was used for centrifugation. Weighting was performed on a calibrated XS205DU analytical balance from Mettler Toledo GmbH (Greifensee, Switzerland). Radioactivity was measured in a calibrated ISOMED 2010 dose calibrator from NUVIA Instruments GmbH (Dresden, Germany). For accurate quantification, small samples (lower radioactivity) were weighted on an analytical balance and counted for 1 min in a 2470 Wizard^2^™ Automatic Gamma Counter (PerkinElmer, Waltham, MA, USA).

### Procedure for preparation of the ^99m^Tc-spiked liquid resin monomer

Pilot experiments were directed towards extraction of technetium-99m from aqueous [^99m^Tc]pertechnetate solutions into the organic solvent *n*-butyl acetate, which could then be mixed with the organic liquid resin monomers for 3D printing. To this end, 500 μL of a diluted [^99m^Tc]pertechnetate solution in 0.9% aq. NaCl (ca. 100 MBq) was mixed with *n*-butyl acetate (490 μL) and trioctylphosphine (10 μL) in a microcentrifuge tube, then thoroughly mixed on a vortex, followed by separation of the phases by centrifugation. Samples from each layer were retrieved, weighted, and the radioactivity measured in a dose calibrator. The data was used to calculate the radioactivity concentrations in both layers and the concentration ratio. Control experiments were conducted in the absence of trioctylphosphine.

**Fig. 1:**
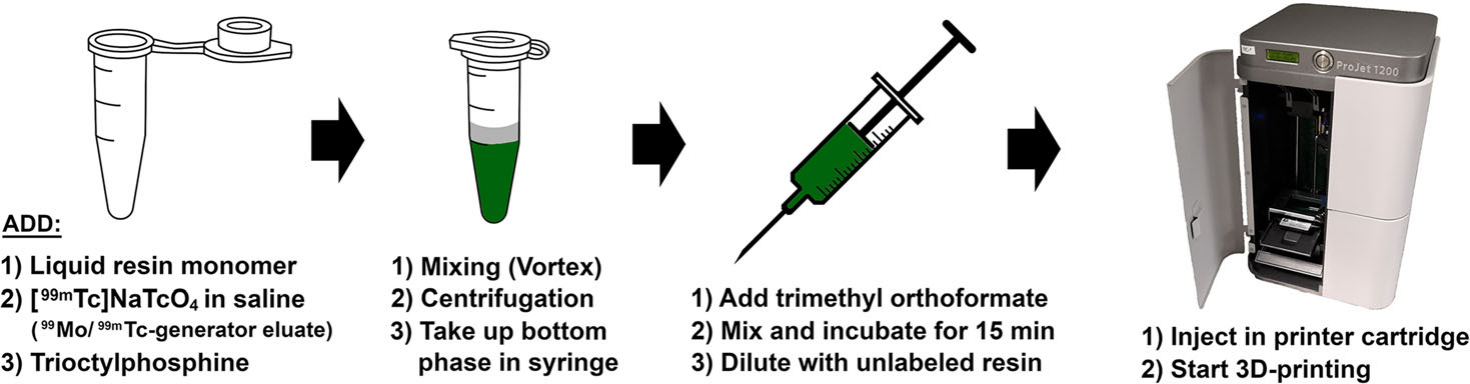
Schematic overview of the procedure for preparation of the ^99m^Tc-spiked liquid resin monomer

Further optimizations led to an improved procedure for direct extraction of technetium-99m from the ^99^Mo/^99m^Tc-generator eluate into the organic liquid resin monomer for 3D printing (Fig. 1). In this procedure, approx. 500 μL of liquid resin monomer was added to pre-weighted 1.5 mL microcentrifuge tube, followed by [^99m^Tc]pertechnetate in 0.9% aq. NaCl (~ 1 GBq, corresponding to 200–300 μL, depending on the radioactivity concentration) and trioctylphosphine (10 μL). The tube was then thoroughly mixed on a vortex (3 × 10 s), followed by centrifugation for 3 min at 16,300×*g*. For calculation of the radioactivity concentrations, the radioactivity of weighted samples from each layer was determined in a dose calibrator. For 3D printing, the ^99m^Tc-spiked resin monomer at the bottom of the extraction tube was carefully taken up in a syringe with a blunt needle (volume of syringe is 10 mL or 15 mL depending on the required final volume for printing). To avoid the risk of taking up part of the remaining water layer, only about 80–90% of the ^99m^Tc-spiked resin monomer was transferred to the syringe. Any possibly remaining residual traces of water in the radioactive resin monomer were chemically removed by addition of trimethyl orthoformate (50 μL) to the radioactive resin in the syringe. After 15 min and repeated mixing, the ^99m^Tc-spiked resin monomer was diluted in the syringe with unlabeled resin monomer to the final volume required for printing. The diluted radioactive resin monomer was thoroughly mixed while taking care not to generate air bubbles and transferred to the printer cartridge. To verify the homogeneous distribution of radioactivity in the resin monomer mixture, samples were taken during transfer of the monomer mixture from the syringe to the printer cartridge, weighted, and the radioactivity measured in a γ-counter. The cartridge containing the radioactive resin monomer mixture (~ 10 g/850 MBq for the bar-shaped phantom; ~ 15 g/630 MBq for the sphere) was then inserted into the 3D printer and used to print the phantom. A schematic overview of the procedure for preparation of the ^99m^Tc-spiked liquid resin monomer is depicted in Fig. 1.

### 3D printing of radioactive phantoms and extractable radioactivity

We initially printed a flat bar-shaped phantom (100 × 20 × 2 mm^3^) to assess possible de-mixing effects during the ~ 7 h lasting printing process, which would result in ^99m^Tc-enrichment or depletion in the residual monomer solution and concomitant variations in the 2D radioactivity distribution in the printed phantom along the printing direction. Following evaluation of the flat-bar shaped phantom, we printed a spherical phantom (diameter 25 mm) to study the 3D radioactivity distribution. The sphere was supported by a non-radioactive stalk, which was printed from non-radioactive resin and glued onto the radioactive sphere.

#### Post-printing treatment

After completion of the printing procedure, the printed objects were rinsed twice with 2-propanol following the procedure described in the user guide using fresh solvent for each washing cycle [17]*.* After drying, the print platform with the printed object was positioned in the UV curing chamber and post cured for about 10 min. The radioactivity of the printed objects was measured in a dose calibrator.

#### Extractable radioactivity

Since a wipe test for surface detachable radioactivity using an ethanol-wetted swap was positive, the radioactive phantom was extracted with 96% ethanol at 50 °C for 1 h. This procedure was done in a closed 50-mL centrifuge tube filled to the maximum with 96% ethanol. The extraction was repeated twice more in new tubes with fresh 96% ethanol. The radioactivity of the three tubes with the ethanolic extracts was quantified in a dose calibrator and in a γ-counter (several samples from each extraction, weight determined on balance). A second wipe test showed only negligible surface detachable radioactivity, necessitating no further extractions. To gain more information on the nature and stability of radioactivity incorporation in the phantom, however, we also investigated potential leaking of radioactivity under acidic (0.1 N aqueous HCl) and basic (0.1 N aqueous NaOH) conditions. The acidic and basic extractions were only single extractions at 50 °C for 1 h and were performed after the extractions with ethanol. For the spherical phantom, an identical extraction procedure was carried out but without acidic and basic extraction.

### Analysis of radioactivity distribution with γ-scanning device and SPECT imaging

Radioactivity distribution in the flat bar-shaped phantom (100 × 20 × 2 mm^3^) was measured on a miniGITA Dual radio-TLC system (Elysia-Raytest, Straubenhardt, Germany) using an OFA probe (V-shaped BGO crystal in combination with PMT tubes) and a 10-mm tungsten collimator (suitable for nuclides 60–250 keV). Detector distance was 4 mm, and measuring time was 10 min. The 2D radioactivity distribution was also recorded on a BrightView X camera (Philips Healthcare) equipped with a low energy high resolution (LEHR), parallel hole collimator. A static acquisition of 0.5 h duration (5.8 × 10^6^ counts) was done. The matrix size was 256 × 256 resulting in a final pixel size of 2.332 × 2.332 mm^2^. The total radioactivity of the phantom (4.82 g) at the start of the γ-camera measurements was 38.27 MBq, resulting in a radioactivity concentration of 7.94 MBq/g. Finally, after completion of all measurements, five small pieces (~ 100 mg per piece) were removed at different positions from the radioactive phantom and put into pre-weighted tubes for γ-counting.

For the spherical phantom (diameter 25 mm), 3D radioactivity data was obtained from SPECT/CT scans acquired on a Discovery NM/CT 670 Pro system (GE Medical Systems). Low dose non-enhanced CT was performed for co-registration and attenuation correction (120 kVp, 93 mAs, pitch factor 0.56). The SPECT/CT scanner was equipped with a LEHR parallel hole collimator. Imaging was done with two detectors 180 ° per detector, 90 projections each, in step and shoot mode, with a circular orbit and 500 s per view in a 256 × 256 matrix. The energy window was set to 140.5 keV for the photopeak with a symmetric energy window of 20% (126–154 keV) and with an additional scatter window of 10% (114–126 keV) width at 120 keV. The phantom data were reconstructed and analyzed on a GE Xeleris workstation (v. 3.1108) using an iterative reconstruction with resolution recovery, attenuation, and scatter correction (OSEM, 3 iterations, 8 subsets). The matrix was 256 × 256, and the final pixel size was 2.209 × 2.209 mm^2^ with 2.21 mm slice thickness. The total radioactivity of the phantom (9.77 g) at the start of the SPECT/CT measurements was 1.28 MBq, resulting in a radioactivity concentration of 0.13 MBq/g.

## Results

### Preparation of ^99m^Tc-containing liquid resin monomer

The basic challenge in the preparation of radioactive liquid resin monomers for 3D printing is the transfer of the radionuclide species from aqueous solutions into the hydrophobic liquid resin monomers. In initial experiments, we investigated the extraction of technetium-99m from aqueous [^99m^Tc]pertechnetate solutions into the organic solvent *n*-butyl acetate, which could then be mixed with the hydrophobic liquid resin monomers. We found that transfer of technetium-99m into the organic phase requires addition of the complex forming additive trioctylphosphine, as > 99% of total radioactivity was transferred to the organic phase in the presence of minute amounts of trioctylphosphine, while essentially all radioactivity remained in the water layer in the absence of trioctylphosphine.

Subsequent optimization experiments led to a simplified procedure for direct extraction of technetium-99m from the aqueous ^99^Mo/^99m^Tc-generator eluate into the organic liquid resin monomers, bypassing the intermediate extraction into an organic solvent (Fig. 1). The optimized method only involves mixing of the aqueous ^99^Mo/^99m^Tc-generator eluate with the organic liquid resin monomers in the presence of a minute amount of trioctylphosphine as a phase transfer agent, followed by separation of the phases. After addition of a small amount of trimethyl orthoformate to remove traces of water, the radioactive liquid resin monomer was thoroughly mixed with the required volume of non-radioactive liquid resin monomer and transferred to the printer cartridge for 3D printing. The printing with the radioactive resin was performed in the same way as with the non-radioactive resin.

Our optimized extraction method with trioctylphosphine proved extremely efficient, as the observed radioactivity concentration ratio between the organic versus the aqueous phase was always > 2000:1 (*n* = 5). Interestingly, transfer of technetium-99m was also observed in the absence of trioctylphosphine, but in this case the radioactivity concentration ratio was only ≥ 10:1 (*n* = 3). Evidently, even a minute amount of trioctylphosphine leads to a substantially improved radioactivity transfer into the organic phase, providing a clear rationale for including this additive in the optimized extraction procedure.

Using our optimized procedure with trioctylphosphine, most of the radioactivity from the aqueous ^99^Mo/^99m^Tc-generator eluate could be transferred into the organic resin monomer in the printer cartridge, apart from some inevitable losses during handling and phase separation. The radioactivity was homogeneously distributed in the liquid resin monomer, as evidenced by γ-counting of weighted samples withdrawn during filling of the printer cartridge with the radioactive resin. The relative standard deviation (RSD) of sample radioactivity concentration was < 2% (*n* = 5).

### 3D printing and assessment of extractable radioactivity

Printing of the flat bar-shaped phantom proceeded smoothly, we did not notice any difference compared to printing with the original cartridges containing the non-radioactive liquid resin monomer. After printing, rinsing, drying, and post curing—normal procedures in 3D printing—the flat bar-shaped phantom was tested for surface detachable radioactivity by carrying out a wipe test with an ethanol-wetted swap. Since the test was positive, three sequential extractions were performed with ethanol at 50 °C (1 h per extraction) to remove any loosely bound radioactivity from the surface of the phantom. The extractable radioactivity (expressed as percentage of the total radioactivity of the phantom) decreased successively from < 1% in the first extraction to < 0.3% in the second and < 0.1% in the third extraction. The ethanolic extractions turned out to be effective in reducing the surface removable radioactivity down to the limit of detection. The radioactive phantom proved also stable towards extraction in acidic and basic aqueous environments, with extractable radioactivities below 0.4‰ and 0.2‰, respectively.

Following evaluation of the flat bar-shaped phantom (large surface-to-volume ratio), we printed a spherical phantom (small surface-to-volume ratio). Under the same conditions (50 °C, 1 h per extraction) and compared to the flat bar-shaped phantom, the ethanol-extractable radioactivity of this phantom was much lower, amounting to only < 0.3% in the first extraction and less than 0.1‰ in the third extraction. Further analysis of the data obtained with both phantoms revealed that the extractable radioactivity is proportional to the surface-to-volume ratio of the phantom, suggesting that radioactivity is stably immobilized in the phantom core and only extracted from a thin layer on the outer surface of the phantom. For illustrative purposes, based on the extraction data and the volume and surface of the phantoms, the calculated thickness of such a surface layer completely devoid of radioactivity is the same for both phantoms and equals only 12 μm.

### Evaluation of 2D and 3D radioactivity distribution in the phantoms

**Fig. 2:**
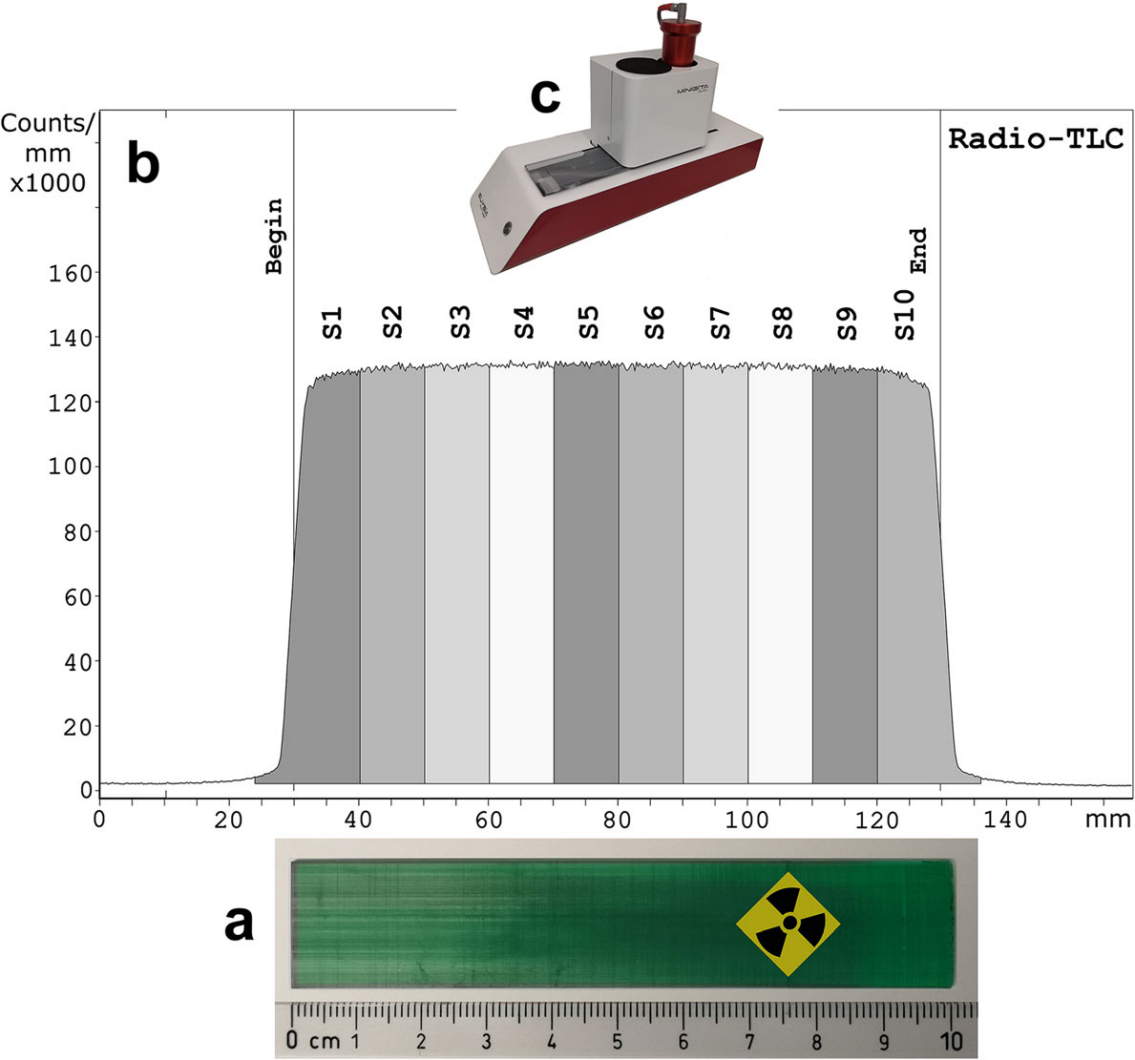
Photo of the green bar-shaped phantom (**a**) and radioactivity distribution along the phantom (**b**) as determined on a radio-TLC system (**c**). Integration of radioactivity counts for each of the ten 1-cm-sections (S1–S10) on the TLC system resulted in almost identical values (~ 1.3 × 10^6^ counts) with a relative standard deviation (RSD) of 0.5%

**Fig. 3:**
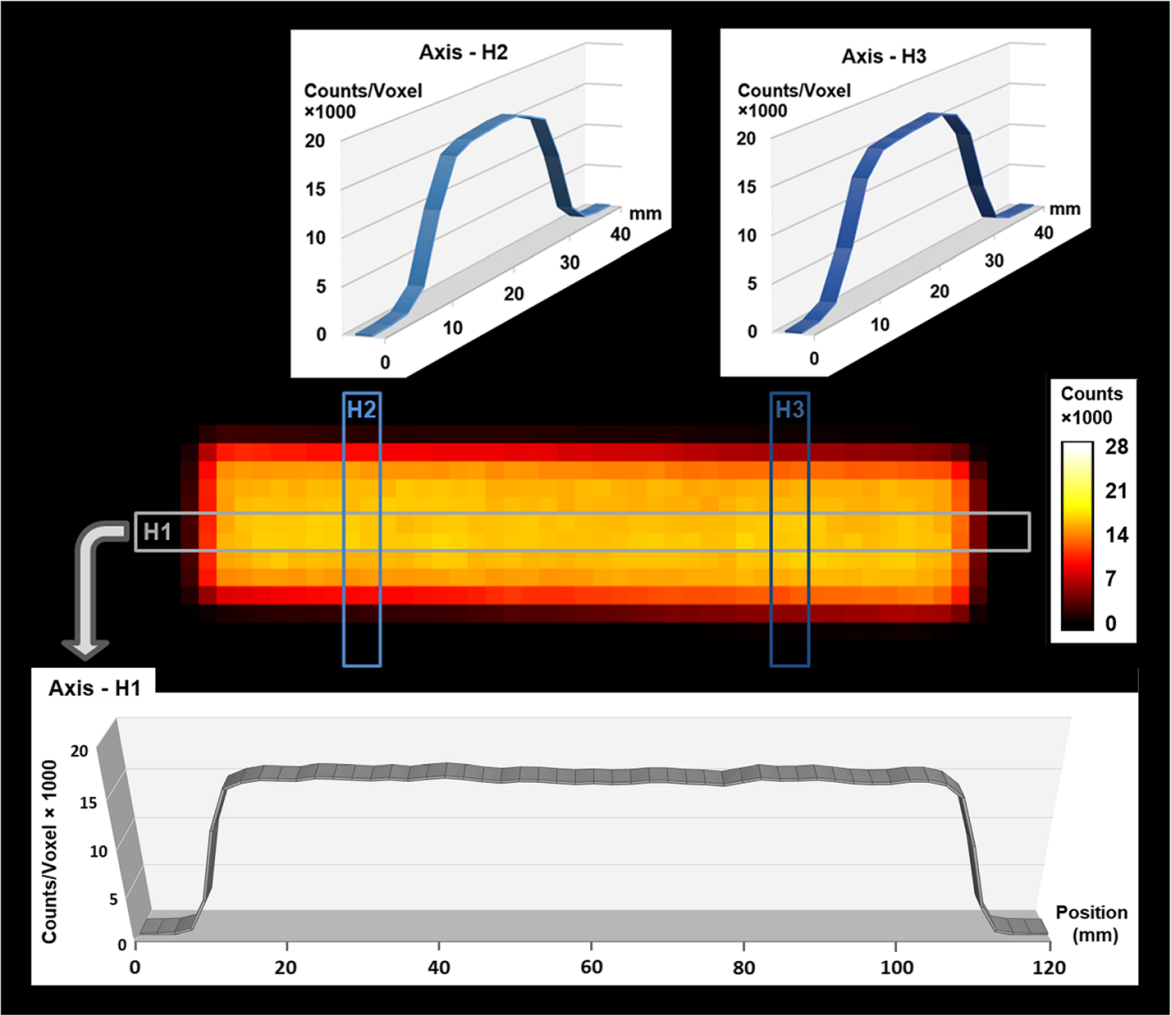
Radioactivity distribution profiles in different directions across the bar-shaped phantom as determined on a γ-camera

The radioactivity distribution profile (Fig. 2b) of the flat bar-shaped phantom (Fig. 2a) was determined on a radio-TLC system (Fig. 2c). The radioactivity was found to be homogeneously distributed along the longitudinal direction, as evidenced both by simple visual inspection of the graph and by integration of the activity in the ten 1-cm-sections S1–S10 (Fig. 2b). In addition, the bar-shaped phantom was also scanned on a γ-camera (Fig. 3). Analysis of the 2D data confirmed the uniform radioactivity distribution in the longitudinal direction (Fig. 3, bottom) and additionally showed that distribution was also homogeneous in the lateral direction (Fig. 3, top). The latter was more or less expected due to the nature of the printing process, where successive layers are built along the longitudinal direction, i.e., the printing material in a lateral layer is added at the same time.

After successful completion of the non-destructive analyses described above, the bar-shaped phantom was cut into small pieces for destructive analysis by γ-counting. The radioactivity concentration of different pieces taken from very different positions of the phantom was essentially the same (RSD ~ 1.2%; *n* = 5).

**Fig. 4:**
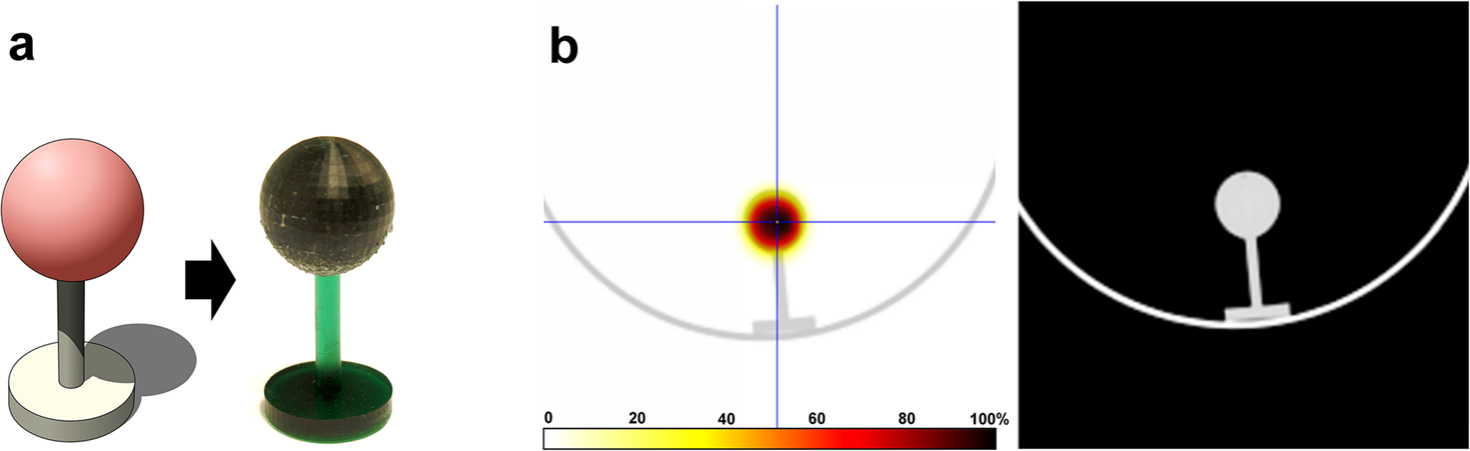
Computer model and photo of the 3D printed spherical phantom (**a**) and the corresponding SPECT/CT images of the radioactive phantom (**b**); note that the pedestal was printed with non-radioactive resin

**Fig. 5:**
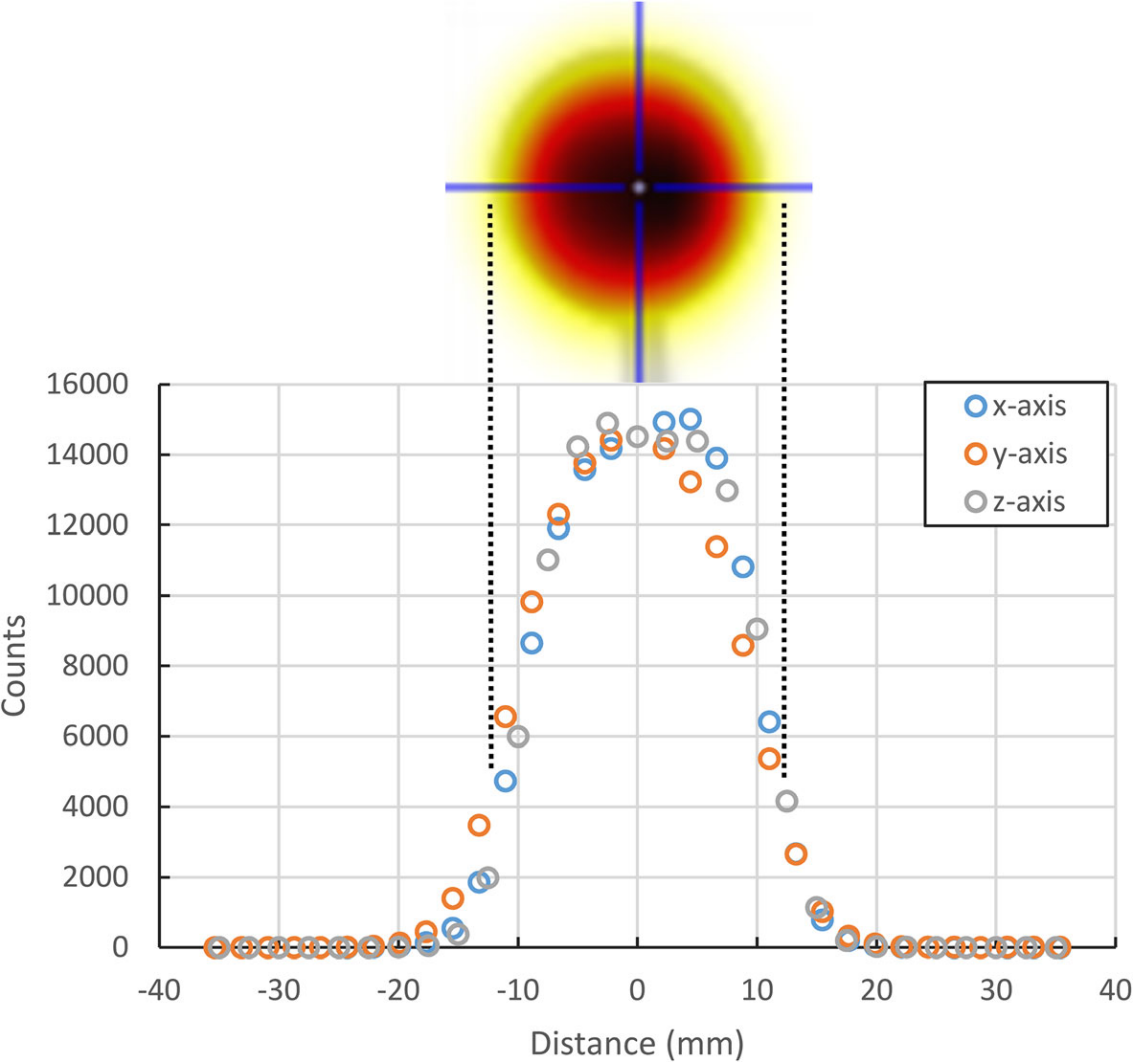
Quantitative analysis of radioactivity concentration profiles along three perpendicular axes through the 3D printed spherical phantom

In a next step, we extended our measurements from 2D to 3D radioactivity data sets using a spherical ^99m^Tc-spiked 3D printed phantom and SPECT/CT scanning (Fig. 4). The axial slice through the sphere’s center displays the typical image of a homogeneous spherical activity distribution. Another indication for the homogenous activity distribution are the similar profiles along all main axes (Fig. 5). The sphere’s CT density of 139.22 ± 2.64 HU corresponded to that of polymethylmethacrylate (PMMA) [19] and was determined in a centered spherical VOI with a volume of 2 cm^3^.

## Discussion

In this feasibility study, we have demonstrated for the first time 3D printing of radioactive phantoms using liquid resin monomers (“3D printer ink”) blended with a suitable radionuclide complex. The choice of technetium-99m for the radionuclide in this study was based on its ease of availability in the clinical environment and its convenient half-life (~ 6 h), which allows printing and subsequent measurements on the phantom, but does not suffer from the disadvantages of radionuclides with longer half-lives, such as high contamination risk and long-lived radioactive waste.

For the transfer of technetium-99m from the aqueous pertechnetate generator eluate into the organic liquid resin monomer, we have developed a customized trioctylphosphine-based method building on the well-known complexation of technetium with phosphine ligands. Cationic ^99m^Tc-complexes of bis(tertiary phosphine) ligands have already been under investigation as potential myocardial imaging agents since the early eighties of the last century [20, 21], and further optimization has led to the development of [^99m^Tc]Tc-tetrofosmin [22], which had received FDA approval in 1996 and is marketed under the trade name Myoview™ [23]. Important insights on the structure of Tc-phosphine complexes were gained from studies of the complexes formed by reduction of pertechnetate with the prototypical bidentate phosphine ligand DMPE [1,2-bis(dimethylphosphino)ethane] [24]. Depending on the reaction conditions, three distinct cationic complexes containing Tc(V), Tc(III), and Tc(I) centers with octahedral coordination geometry were obtained [25]. In the course of their search for small, water-soluble and air-stable phosphine ligands for use as bifunctional chelating agents (BFCAs), Berning and colleagues [26] investigated the ^99m^Tc-complex of *tris*(hydroxymethyl)phosphine (THP), which is presumably a Tc(V) species resulting from reduction of pertechnetate [Tc(VII)] and concomitant oxidation of the THP. The ^99m^Tc-THP-complex can be prepared by simply mixing the ^99^Mo/^99m^Tc-generator eluate with a solution of THP in saline. THP is provided in excess and has a dual role both as reducing and as complexing agent.

Inspired by the facile preparation of the highly hydrophilic [^99m^Tc]Tc-THP complex (chloroform-water partition coefficient < 0.0001) [26], we evaluated the structurally closely related lipophilic trioctylphosphine (TOP) for transfer of technetium-99m from the aqueous ^99^Mo/^99m^Tc-generator eluate into the organic acrylate-based liquid resin monomer. The extraction of technetium-99m into the organic liquid resin monomer was very efficient, suggesting formation of a stable [^99m^Tc]Tc-TOP complex comparable to the [^99m^Tc]Tc-THP complex (*vide supra*). We demonstrated that this TOP-complexed technetium-99m is homogeneously distributed and stably immobilized in the 3D-printed phantom, necessitating no further efforts towards improvement of the ligand, such as, e.g., by developing customized phosphine ligands with acrylate side chains to covalently anchor the ligands in the resin by copolymerization.

While technetium-99m is an interesting radionuclide for certain SPECT-applications, we expect that future developments in the field will focus on solid-state phantoms containing radionuclides with longer half-lives, in particular positron emitters for PET, which can be used for site qualification in multicenter clinical trials and for longitudinal measurements on a single PET/CT scanner [27]. Building on our experience with 3D-printed ^99m^Tc-phantoms, we are currently developing customized procedures for 3D printing of phantoms containing radionuclides with longer half-lives (*t*_½_), such as germanium-68 (*t*_½_ = 271 days) for PET- and cobalt-57 (*t*_½_ ~ 272 days) for SPECT applications. Following the successful proof-of-concept for the production of small phantoms, our ultimate goal is the production of larger phantoms, which are typically used in SPECT and PET. Apart from potential challenges regarding radiation protection and provided that a suitable 3D printer for printing larger objects is available, we do not foresee major complications, since our procedure for preparation of the radioactive liquid resin monomers (“3D printer ink”) can easily be upscaled by simply using a higher amount of radioactivity for the extraction followed by dilution into a larger volume of liquid resin monomers.

Once the basic methodologies for incorporation of various radionuclides into the monomeric printer resins have been established, we also envisage future research efforts towards 3D printing of “multicolored” phantoms, i.e., phantoms containing substructures with different radionuclides in different concentrations. Evidently, these phantoms cannot be produced on 3D printers using a liquid resin bath and Digital Light Processing (DLP) methods, such as the ProJet® 1200 printer. Instead, Multi-Jet Printing (MJP) or Poly-Jet Printing (PJP) techniques may be the methods of choice [7]. With these techniques, the 3D model is built layer-by-layer by deposition of photopolymer materials followed by curing with UV light. In principle, the composition of the printing material deposited at a given moment may be controlled by in situ mixing of different standard materials (different types of liquid resin monomers and/or additives) in a suitably designed multi-material printing head [8]. In this way, complex phantoms with local variations of resin materials can be produced, including different concentrations of one or more radionuclides and/or additives to control the radiodensity.

## Conclusions

We have developed a procedure for incorporation of technetium-99m into liquid resin monomers for 3D printing and demonstrated for the first time the successful application of this methodology for printing of radioactive phantoms. The 3D printed radioactive phantoms are characterized by a well controllable, homogeneous distribution of radioactivity, which is stably bound in the resin. Compared to conventional mold phantoms filled with radioactive liquids, 3D printed radioactive phantoms can be produced in almost any shape and without cold walls. While the scope of the present study is limited to the relatively short-lived radionuclide technetium-99m, we anticipate that similar procedures will eventually allow the preparation of 3D printed solid phantoms containing long-lived radionuclides. Such phantoms will lead to improvements in scanner validation procedures and can serve as a standard in multicenter trials, since they avoid the error-prone preparation of liquid-based phantoms at different centers.

## Data Availability

The data generated and analyzed in this study are available from the corresponding author on reasonable request.

## Abbreviations

BGO: Bismuth germanium oxide
CT: Computed tomography
DLP: Digital light processing
HU: Hounsfield units
LED: Light emitting diode
LEHR: Low energy high resolution
MRI: Magnetic resonance imaging
OFA: “One-fits-all”
OSEM: Ordered subset expectation maximization
PET: Positron emission tomography
PMMA: Polymethylmethacrylate
PMTs: Photomultiplier tubes
RSD: Relative standard deviation
SPECT: Single-photon emission computed tomography
*t*_½_: Physical half-life
THP: *Tris*(hydroxymethyl)phosphine
TOP: trioctylphosphine
US: Ultrasound
VOI: Volume-of-interest

## References

[1] Clarke BN (2018). PET Radiopharmaceuticals: what’s new, what’s reimbursed, and what’s next?. J Nucl Med Technol..

[2] Vasdev N, Alavi A. Issue “Novel PET Radiotracers with Potential Clinical Applications.”. PET Clinics. 2017;12 i-x and 269-372. 10.1016/j.cpet.2017.04.002 28576174

[3] Sharma P, Mukherjee A (2016). Newer positron emission tomography radiopharmaceuticals for radiotherapy planning: an overview. Ann Transl Med..

[4] Reischl G (2019). Special Issue “Targets, Tracers and Translation Novel Radiopharmaceuticals Boost Nuclear Medicine.”. Pharmaceuticals (Basel).

[5] Doot RK, Pierce LA, Byrd D, Elston B, Allberg KC, Kinahan PE (2014). Biases in multicenter longitudinal PET standardized uptake value measurements. Transl Oncol..

[6] Gosewisch A, Ilhan H, Vomacka L, Böning G (2018). Dosimetrie bei der Radionuklidtherapie mit Lu-177. Nuklearmediziner..

[7] Kim GB, Lee S, Kim H, Yang DH, Kim Y-H, Kyung YS (2016). Three-dimensional printing: basic principles and applications in medicine and radiology. Korean J Radiol..

[8] Wang K, Ho C-C, Zhang C, Wang B (2017). A review on the 3D printing of functional structures for medical phantoms and regenerated tissue and organ applications. Engineering..

[9] Filippou V, Tsoumpas C (2018). Recent advances on the development of phantoms using 3D printing for imaging with CT, MRI, PET, SPECT, and ultrasound. Med Phys..

[10] Valladares A, Beyer T, Rausch I. Physical imaging phantoms for simulation of tumor heterogeneity in PET, CT, and MRI: an overview of existing designs. Med Phys. 2020. 10.1002/mp.14045. 10.1002/mp.14045 PMC7216968 31981214

[11] Hofheinz F, Dittrich S, Pötzsch C, van den Hoff J (2010). Effects of cold sphere walls in PET phantom measurements on the volume reproducing threshold. Phys Med Biol..

[12] Larsson SA, Jonsson C, Pagani M, Johansson L, Jacobsson H (2000). A novel phantom design for emission tomography enabling scatter- and attenuation-“free” single-photon emission tomography imaging. Eur J Nucl Med..

[13] Van Laere KJ, Versijpt J, Koole M, Vandenberghe S, Lahorte P, Lemahieu I (2002). Experimental performance assessment of SPM for SPECT neuroactivation studies using a subresolution sandwich phantom design. Neuroimage..

[14] Holmes RB, Hoffman SMA, Kemp PM (2013). Generation of realistic HMPAO SPECT images using a subresolution sandwich phantom. Neuroimage..

[15] Negus IS, Holmes RB, Jordan KC, Nash DA, Thorne GC, Saunders M (2016). Technical note: development of a 3D printed subresolution sandwich phantom for validation of brain SPECT analysis. Med Phys..

[16] Berthon B, Marshall C, Holmes R, Spezi E (2015). A novel phantom technique for evaluating the performance of PET auto-segmentation methods in delineating heterogeneous and irregular lesions. EJNMMI Phys..

[17] ProJet^TM^ 1200 User Guide. [cited 2019 Dec 5]. Available from: http://infocenter.3dsystems.com/product-library/sites/default/files/printers/projet1200/2515_341378-00_RevF_UserGuide.pdf.

[18] Safety Data Sheet VisiJet® FTX Green (rev. Sept 2014). [cited 2019 Dec 5]. Available from: http://infocenter.3dsystems.com/materials/sites/default/files/sds-files/professional/341600-s12-01-a-sds-ghs-english-visijet-ftx-green.pdf.

[19] Schneider W, Bortfeld T, Schlegel W (2000). Correlation between CT numbers and tissue parameters needed for Monte Carlo simulations of clinical dose distributions. Phys Med Biol..

[20] Deutsch E, Glavan KA, Sodd VJ, Nishiyama H, Ferguson DL, Lukes SJ (1981). Cationic Tc-99m complexes as potential myocardial imaging agents. J Nucl Med..

[21] Deutsch E, Bushong W, Glavan KA, Elder RC, Sodd VJ, Scholz KL (1981). Heart imaging with cationic complexes of technetium. Science..

[22] Kelly JD, Forster AM, Higley B, Archer CM, Booker FS, Canning LR (1993). Technetium-99m-tetrofosmin as a new radiopharmaceutical for myocardial perfusion imaging. J Nucl Med..

[23] Woolley GT, Kitson SL, Reid RG (2007). Synthesis of the myoview^TM^ ligand, [bisphosphinoethane-1,2-14C]tetrofosmin. J Label Compd Radiopharm..

[24] Deutsch E (1993). Aspects of the chemistry of technetium phosphine complexes. Radiochim Acta..

[25] Vanderheyden JL, Ketring AR, Libson K, Heeg MJ, Roecker L, Motz P (1984). Synthesis and characterization of cationic technetium complexes of 1,2-bis(dimethylphosphino)ethane (DMPE). Structure determinations of trans-[TcV(DMPE)2(OH)(O)](F3CSO3)2, trans-[TcIII(DMPE)2Cl2]F3CSO3, and [TcI(DMPE)3]+ using x-ray diffraction, EXAFS, and technetium-99 NMR. Inorg Chem..

[26] Berning DE, Katti KV, Singh PR, Higgenbotham C, Reddy VS, Volkert WA (1996). In vitro and in vivo characterization of a 99mTc complex with tris(hydroxymethyl)phosphine (THP). Nucl Med Biol..

[27] Prenosil GA, Hentschel M, Fürstner M, Krause T, Weitzel T, Klaeser B (2017). Technical note: transconvolution based equalization of positron energy effects for the use of 68 Ge/68 Ga phantoms in determining 18 F PET recovery. Med Phys..

